# The role of substance use in the lives of incarcerated older adults: A qualitative study

**DOI:** 10.3389/fpsyt.2023.1116654

**Published:** 2023-03-13

**Authors:** Hila Avieli

**Affiliations:** Department of Criminology, Ariel University, Ari'el, Israel

**Keywords:** incarcerated older adults, drug abuse, life course perspective, interpretive phenomenological analysis, incarceration and health

## Abstract

**Background:**

The prevalence of drug abuse among older adults has grown over the last decade. Despite the expanding development of a body of research dedicated to studying this phenomenon, drug abuse by incarcerated older adults has been marginalized. Thus, the aim of the present study was to explore drug abuse patterns in the lives of incarcerated older adults.

**Method:**

Semi-structured interviews were conducted with 28 incarcerated older adults, and an interpretive analysis was used to analyze the participants’ narratives.

**Findings:**

Four themes emerged: (1) Growing up around drugs; (2) Prison onset; (3) Professionals, and (4) Lifelong substance abuse.

**Conclusion:**

The study findings reveal a unique typology of drug-related themes in the lives of incarcerated older adults. This typology sheds light on the interplay between aging, drug use, and incarceration and the way these three socially marginalized positions may intersect.

## Introduction

The worldwide number of incarcerated older adults (IOAs) has increased over the past few decades, reflecting the growing aging population (1). Correspondingly, the past decade has seen a demonstrable shift in the age profile of individuals using drugs (2). An aging cohort, who has either survived lengthy histories of drug use or has just started abusing drugs, currently accounts for an increasing proportion of those in need of drug abuse treatment (2–4). Notwithstanding the incipient development of a body of research dedicated to studying older adults’ drug use, the phenomenon of IOAs’ drug abuse has been marginalized (5). Moreover, specific research that qualitatively examines subjective accounts of drug abuse among IOAs is lacking. This type of evaluation is critical given the complex and nuanced evidence regarding the exclusive characteristics of drug abuse among these individuals (6).

### Older adults and drug abuse

Traditionally, it was assumed that drug abuse diminished during adulthood (7). However, cumulative evidence has challenged this preconception, suggesting that some people continue to use drugs well into their aging years (2). The trend of older adults’ drug abuse is associated with the prevalence of addiction to prescribed medication within this group (2, 8). It is also associated with the coming of age of individuals who grew up during the 1960s and 1970s—a period of changing attitudes toward drug and alcohol use (9). Another factor linked to this trend relates to changes in the treatment management of opiates during the 1990s (10), namely the use of methadone therapy that led to improved survival rates among people who abuse drugs (11). Despite the rapidly increasing literature concerning older adults’ drug use, there is less research on the subject among prison populations (5, 12). In a recent scoping review on older adults who use illicit opioids, cocaine, or methamphetamine (13), out of 164 papers included in the review, only 15 mentioned participants’ incarceration as part of the study, often referring to incarceration history only when describing the study population or when distinguishing between comparison groups. Similarly, another review by Maschi and Dasarathy (14) concluded that there was a significant unexplored research and practice gap in the literature concerning justice-involved older adults with respect to the prevalence of mental health disorders including drug abuse and other addictions. Thus, the aim of the present study was to target this gap by addressing the role of substance use in the lives of incarcerated older adults.

### Incarcerated older adults and drug abuse

As noted, limited studies have examined IOAs coping with drug abuse issues (15). Previous research has suggested that IOAs are atypical in their drug abuse habits (16), meaning that although IOAs tend to use more drugs than their age peers living in the community (6), their drug use is markedly lower than that of younger incarcerated individuals (17). In addition, alcohol abuse is the most prevalent substance used by IOAs (5), followed by opioids (18). This contrasts the preference for cannabis that is prevalent among incarcerated individuals who use drugs in general (19). The literature shows that drug use is generally perceived as being a problem of younger incarcerated individuals and, therefore, treatment services are usually designed to cater to the needs of the younger population rather than of IOAs (20, 21). Some IOAs, however, may have a very lengthy substance use career and may have difficulty envisaging the rest of their lives without drugs. Thus, it is surprising that substance abuse treatment programs are rarely offered to IOAs (5, 22). In addition, substance abuse was among the most frequently reported health problems for older offenders according to Solares et al. (23); particularly, use of alcohol, opioids, and cannabis is associated with functional limitations and disability (24). While most existing studies aim to map the landscape of IOAs’ drug abuse, pinpointing primarily epidemiological questions, e.g., How many IOAs use drugs? Which substances are used? (13, 23), the experiences of IOAs who use drugs have received less attention. These experiences were addressed mainly anecdotally, as part of a wider exploration of older adults’ experience of incarceration, or as part of a study of individuals’ substance use experience in prison, when some of the participants happened to be older adults. For example, Shibusawa and Padgett (25) drew a connection between traumatic childhood events, early-onset drug use, and incarceration among formerly homeless adults with chronic mental illness. They focused on the losses caused by substance abuse in the lives of the participants and noted that some of them viewed old age as an opportunity to eliminate substance dependency. Similarly, Pageau (26) found that some IOAs perceived their time in prison as an opportunity to stop consuming drugs. Boeri et al. (27) compared the experiences of early- and late-onset older individuals who use heroin. They found that early-onset drug abuse by IOAs was often exacerbated by either frequent or long periods of institutionalization that hindered their ability to bond with mainstream society. This is consistent with Padgett (28), who brought forth the voices of older adults who had lost contact with their children and other family members as a result of their substance abuse and incarceration periods. However, Pope et al. (29), in their study of former IOAs, noted that the loss of contact with children had led to substance abuse. Moreover, they found that IOAs with drug abuse problems had often been exposed to drug-using parents from early childhood. Finally, Wyse (30) studied the narratives of ex-incarcerated individuals who struggled with lifelong drug addiction. She described prison-enforced detoxification as a turning point that provided the participants in her study with clarity, often followed by a period of reflection and evaluation of their lives. Older age seemed integral for this period of sobriety and reflection to catalyze cognitive change and inspire a new commitment to sobriety. Thus, it seems that narratives related to drug abuse by IOAs may produce a nuanced and complex picture of both the losses and hardships intertwined with healing. Nonetheless, while these findings are compelling, they are still anecdotal.

### Life-course perspective as a theoretical framework for studying the subjective accounts of drug abuse among incarcerated older adults

This analysis is underpinned by the life-course perspective (LCP), which is a multidisciplinary holistic approach used to examine aspects of continuity, change, transition, and stability throughout human lives (31, 32). By focusing on the patterns or trajectories of individuals’ lives and the ways in which those patterns are shaped by the broader historical context and social structures, the life-course perspective offers a broadened organizing framework. It potentially allows for characterizing distinctive patterns of drug use trajectories, identifying critical events and factors contributing to persistence or change during the life span, and analytically ordering the events that occur during that course (33). Some key life-course concepts that may be useful for understanding older adult’s drug abuse experience are trajectories and transitions. A trajectory is a pathway or line of development during the life span, such as work life, parenthood, or criminal behavior. Transitions are short-term changes in stages or roles within that same trajectory (e.g., starting or leaving school, starting to use drugs, committing the first crime). Some, but not all, transitions lead to turning points that produce long-term behavioral change (34). Turning points have typically been described as major life events that may redirect an individual’s life path and lead to changes in life trajectories (35). Indeed, some studies, in which the life-course perspective has been applied to the phenomenon of older adults’ drug abuse, have used these concepts and revealed insightful findings (36). For example, a qualitative study based on interviews with 20 older adults with drug abuse problems suggested that older adult with substance abuse problems tended to self-identify as individuals who transitioned into substance abuse at old age whereas, in fact, most of them had long-term substance abuse problems that were exacerbated by age-related events (37). Another life-course-informed investigation of older adults’ drug abuse revealed that, contrary to expectations, those older individuals who use drugs, who’s addiction has started at younger age, were better able to control their drug use habits as well as to maintain mainstream social roles than those who had started later in life (27). In line with these findings are those of Cepeda et al. (38), which indicate that emerging dysfunctions in the heroin lifestyle of older adults who use drugs lead not to cessation but rather to “maturing in”—a specific process of social readjustment that returns the individual to a stable maintenance pattern of use instead of a recovery phase. Finally, Grella and Lovinger (39) identified distinctive trajectory patterns of drug use over a 30-year period among participants in a methadone treatment program. They found that older adults who had used heroin the longest were characterized by relatively higher levels of childhood conduct problems and adult antisocial personality disorder. They suggested that these longstanding drug habits and criminal involvement may be associated with an underlying childhood psychopathology.

While studies using LCP to explore older adults’ experience of drug abuse have produced meaningful findings, the issue of IOAs’ drug abuse, informed by LCP, seems to be absent from the literature, in keeping with the general absence of research on IOAs’ drug abuse experience.

Thus, the aim of the present study was to investigate the ways in which incarcerated older adults give meaning to their drug abuse experiences along the life course.

The research question was as follows: What was the meaning of drugs and drug abuse in IOAs lives? What were the roles drugs played in their lives? How were drugs and drug abuse experienced before or during their incarceration?

## Methods

An interpretive phenomenological analysis (IPA) method (40) was chosen to analyze the narratives of the participants in current study. This contemporary qualitative methodological approach is committed to the systematic exploration of lived experiences and to understanding how individuals make sense of their personal and social worlds (41). This qualitative approach explores the lived experiences individuals life (41), and it has been used in a wide variety of criminology and criminal justice related studies (42–44), as well as in prison research (45).

### Participants and sample

The research sample included 28 incarcerated males recruited by the state’s Prison Service. Purposive sampling (46) was used to select information-rich cases. The inclusion criteria specified that participants must be over 55, who either have a history of drug use or use drugs currently, and had no other serious health issues at the time of the interview. This was verified by crosschecking several provisions: The participant’s incarceration history sheet showed at least one drug-related crime in his record; the participant had been offered at least one drug abuse treatment or drug-related therapeutic intervention during his incarceration history, and the participant self-identified as a former or current drug abuser. All drug types were accepted, as well as a combination of drug and alcohol abuse, but IOAs who abused alcohol only were excluded from the study. It is important to note that not all participants were diagnosed with substance use disorder – some participants used drugs occasionally or only as younger men, while others had more severe drug use history. An additional criterion was having served at least 3 years in prison (either consecutively or in separate terms). This criterion was included to make sure that only individuals who had accumulated sufficient prison-life experience would take part in the study. The participants’ ages ranged from 55 to 82, with a mean age of 66.8, and a mean number of 13 years’ incarceration. Additional socio-demographic data regarding the participants is presented in Table 1.

**Table 1:** Sociodemographic characteristics of the participants.

Participant number	Age	Number of years served in prison at the time of the interview (Multiple sentences)	Cumulative years of drug usage*	Types of drugs used along the years**
1	73	9	17	Cannabis, Opioids
2	55	8	20	Opioids
3	81	6	60	Cannabis
4	64	31	49	Cocaine, Cannabis, Heroin, Amphetamines
5	79	4	18	Opioids
6	59	6	37	Cocaine, Cannabis, Heroin, Amphetamines
7	66	17	11	Cocaine
8	63	15	20	Heroin
9	57	20	16	Heroin
10	60	7	3	Cannabis
11	61	12	21	Opioids
12	71	4	7	Opioids
13	60	27	46	Heroin, Crack cocaine
14	73	6	10	Cannabis
15	69	24	5	Cannabis
16	68	19	23	Cannabis
17	78	4	4	Cannabis
18	71	9	40	Crack cocaine
19	56	22	36	Heroin
20	82	11	5	Opioids
21	59	14	7	Opioids
22	62	11	27	Heroin, Amphetamines
23	63	6	10	Opioids
24	59	8	18	Cannabis
25	69	5	12	Opioids
26	67	24	30	Heroin
27	70	11	45	Cannabis, Heroin
28	77	25	25	Cocaine, Cannabis, Heroin, Amphetamines

*Overall estimated drug use years including usage intermissions. **Participants were asked to recall only illicit drugs used, not including alcohol and prescription medication.

At the time of the interviews, the participants were housed in three state prisons. Seven participants were living in special rehabilitative wards in a minimum-security prison, nine participants were living in a special ward designated to older adults in a different minimum-security prison, and 12 participants were living in regular wards in a high-security prison. The final sample size was determined according to Morse’s principle of theoretical saturation, which asserts that when repeated content, details, and interpretations emerge across interviews, than data saturation has been reached (47). In the present study, after interviewing 28 participants, saturation has been reached.

### Data collection

Data collection was performed *via* in-depth, semi-structured phenomenological interviews using an interview guide (48) that addressed the following categories: (1) The experience of drug use through the years (e.g., How did you first start using drugs? What was the role of drugs in your life as a young man?); (2) The meaning of drug use in a criminal lifestyle (e.g., What does it feel like when you use drugs in prison? How has that experience changed during your incarceration?); (3) Aging and drug use (e.g., How has aging changed your drug use habits? Looking back at your time in prison, how did it affect your drug use through the years?).

### Procedure

The research was carried out in collaboration with the state’s Prison Service. Following research approval by the University’s Institutional Review Board (IRB) (approval number AU-SOC-HA-20220724) and the approval of the prison service research committee, a social worker from each prison was appointed as a contact person to coordinate the research. They handed out an explanation sheet with the aims and scope of the research, as well as the research criteria, inside the prison wards, asking IOAs who wished to participate to reach out to them or to one of the wardens who were also aware of the research. The social workers or the wardens referred willing participants, who met the research criteria, to the author, and the social workers coordinated interview times and dates. A total of 31 IOAs expressed willingness to participate in the study. However, three of them changed their minds and canceled their interviews. The interviews themselves were conducted inside the prison ward in a private room in the presence of only the researcher and the IOA; no prison guard was present. Each interview usually lasted from1–2 h depending on the participant’s individual needs and communication abilities.

In light of the sensitivity of the participating population—IOAs sharing details about their drug use habits (49)—special provisions were made to ensure informed consent and confidentiality (50). Written informed consent was obtained from each participant. In addition, the author continuously sought to obtain process consent from those who appeared distressed during the interview (51). If difficult emotions were expressed, the author has advocated for crisis management by the prison’s social worker (52). The interviews were audio-recorded and transcribed verbatim. All identifying details were changed to preserve the participants’ confidentiality. The data collected during the study were kept confidential, stored in a locked computer, and protected with a password known only to me.

### Data analysis and trustworthiness

Data analysis was conducted according to the guidelines of the interpretive phenomenological analysis (IPA) approach, as suggested by Smith, Flowers, and Larkin (40). First, the transcripts were read several times so the researcher will become as familiar as possible with the text. Then, inductive initial coding was performed by coding the participants’ significant statements, for example, referring to their wide range of feelings about drug use through their life course. The next step involved grouping the statements into subthemes and identifying quotes that captured the essential quality of the participants’ experiences and perceptions. For example, quotes relating to the way participants viewed the role of drug use in their lives were gathered (e.g., depending on drugs, denying their drug problems, using drugs to advance their criminal lifestyle). The next step included making connections, clustering and conceptualizing them these quotes. At this stage, LCP (31) was used heuristically in a deductive way to provide a broader understanding of the findings and to enable identification of four superordinate themes reflecting different roles illicit substances has played in IOAs lives (40). During the textual analysis, the researcher aimed to identify the varying similarities and differences of participants’ accounts. For instance, participants generally viewed themselves as victims of the prison system. However, some made a connection between drug use and incarceration while ots perceived their drug use as a personal problem rooted in their childhood experiences. (40).

Trustworthiness of the findings was ensured as follows: first, audio-recorded interviews and their verbatim transcriptions enabled verification with the original, for referential adequacy (53, 54). Second, experiences and preconceptions regarding older adults, IOAs, and drug use were bracketed by the researcher, to prevent them from influencing the research or its interpretation (55). This was accomplished as described by Moustakas (56), by engaging in repeated rounds of self-reflection on preconceptions and prejudgments. Moreover, an ongoing reflective journal was used, which enabled the expression of thoughts and feelings. Issues that arose from the reflective journal were discussed with a group of colleagues, thus enabling further bracketing (57).

## Findings

The participants’ narratives revealed four major themes composing different roles of illicit substances in IOAs lives: (1). Growing up around drugs: Drugs as an inherent part of the social and familial childhood landscape; (2). Prison onset: Drug use that started in the prison setting; (3). Professionals: A drug-dealing career; (4). Lifelong substance abuse: Individuals with a substance abuse disorder who have been using drugs for most of their lives.

### Growing up around drugs: Drugs as an inherent part of the social and familial childhood landscape

Some IOAs, who were not involved in drug offences at this point in their lives, mentioned drugs in their personal history:

I was a good kid, kind of a good student, but the streets, they kind of pulled me in, they had this power over me … I would stand outside and look at the cars drive by, at people who have cars, and have stuff, my house was … we had practically nothing… my parents, well they were weak, they had a hard time raising us, so I spent most of my days outside. Sure, I used to do some drugs, got busted a few times, but mostly I loved the action and it was easy for me stop going to school and just hang out … the streets in those days were chaos, crime, violence, prostitutes, and drugs everywhere … drugs were just out in the open, people would buy and sell over coffee shop tables in the sixties, and I, as a teenager, would run errands for those drug dealers. I looked up to them, they were the role models of my childhood … Now this was my school … (IOA, 73).

This participant vividly described the crime and drug industry that was the backdrop to his adolescence. He portrayed the allure of street life as an irresistible force that dragged him in, offering far better opportunities than his family’s empty house. The dichotomy between “full” and “empty” continued when the participant described his parents as weak and the local drug dealers as role models. Although his own substance use history was minor, it is apparent that growing up in an environment overflowing with drugs and drug dealers had shaped his values and provided him with knowledge about the know-hows of the criminal world.

Another participant, who was peripherally involved in drugs as a younger man, explained the meaning of growing up around drugs:

My parents were both addicts; my dad had a serious drinking problem and my mom was hooked on opioids. I guess I've never said that out loud out to anyone except to my social worker here … It took me a while, a lifetime in fact, to grasp the meaning of what it did to us, to me and my brother and sister … we are all screwed up. Today, I know that my whole concept of good, bad, relationships, love, law, is fucked up … my brother and I have been in prison for years now and my sister is dead. Abiding by the law was always, like, just one possibility, not the first choice. And you know, I did some drugs. I was never, like, a serious addict, never dealt drugs, but looking back on all my years of violence, I know now that I am a drug victim … " (IOA, 69).

This participant had undergone intensive psychotherapy during his incarceration and shared his insights regarding his lifelong involvement in crime. He appeared to attribute his tendency to violence and disobedience to growing up with drug-using parents and viewed his and his siblings’ fate as gravely associated with the setting in which they were raised. Specifically, he pinpointed basic life skills, such as differentiating between good and bad, that are usually taught at a young age, either directly or by parents’ personal example. These values were not taught, and profoundly affected the participants’ path into a criminal life.

It is apparent from these narratives that drug abuse, *per se*, was not a major issue in their lives. Nonetheless, the participants reflected on spending their early life in the local drug scene or living with parents who were drug addicts, and perceived these formative experiences as having laid the foundations for their life-long criminal involvement. That is to say, for these participants, drug abuse was a state of mind and a set of values and attitudes that guided them through life. In this context, the life-course perspective (LCP) views the individual’s own developmental path as embedded and transformed by conditions and events that occurred during the historical period and geographical location in which the person lives (31). Here, the participants described growing up at a time when the streets of some cities had a bustling drug scene. These participants viewed growing up in an environment that was socially receptive toward drug use as an inherent part of their criminal career. Some of them also mentioned their parents’ role in their life choices. The life-course principle of linked lives refers to the interdependence and reciprocal connection of people’s lives and explains how individuals in a significant relationship (e.g., parents and children) share influential, merged, and lifelong developmental trajectories (31). This concept may help explain how parental drug abuse can reverberate through the generations, causing the children who are unwitting, vicarious victims to repeat that behavior.

### Prison onset: Drug use that started in the prison setting

Participants whose narratives were part of the current theme started using drugs in incarceration, usually when they were in their thirties or forties. These participants described drug use as a lifeline in the grim reality of prison life:

So, I moved around quite a lot from prison to prison during my sentence. I started at one prison and then after 9 years moved to another, and then moved again … This is my fifth place. I always had problems with the other guys and spent a lot of my time in prison in isolation. You know what that means? Being in isolation for months and months? I started using in my forties. After I tried to kill myself, they wouldn't pull me out of isolation, but I could see people at group therapy and in the yard and they helped me get some stuff. I owe them my life because drugs were the only thing that can get a person through a long incarceration. Without those drugs, I would probably have tried to kill myself again … (IOA, 65).

After years of drug-free incarceration, this participant viewed his introduction to the world of drugs as a pathway to liberation within confinement. He voiced the pain and loneliness of being tossed around within the prison system, facing social difficulties, and spending most of his time completely alone. The participant drew attention to the irony of the situation: It was only when he attempted suicide that he received some therapeutic help. The therapy itself did not work, but gave him better access to drugs.

Another IOA described as follows:

A life sentence, with no parole in sight, my case is not discussed anywhere, it's like, something you learn to live with … looking back from where I am now, one of the things that got me through was getting immersed in prison life, trying to forget that there is a world outside, bringing, like, all my energies to this place … I became really close with some major players here, they showed me the ropes… first cocaine, then heroin, then whatever was available … I did it without even thinking. It was part of prison life, plain and simple … they all left after 10 years, 15 years. I'm still here … (IOA, 77).

This participant described drug involvement as part of a “package deal” for individuals incarcerated for long periods of time. For him, surviving prison was built on engaging socially with other incarcerated individuals and becoming involved in prison life. This involvement included immersion into the drug scene almost carelessly: “I did it without even thinking.” He did, however, appear to feel somewhat deceived while revealing that all his good friends, who had drawn him into the world of drug use, had eventually been released, while he remained in prison.

The offset of drug abuse while incarcerated echoes the LCP perception of trajectories, transitions, and turning points (58). In the present study, the participants narrating this theme had been involved in a drug-free criminal lifestyle (a trajectory). Aging within a criminal lifestyle had summoned new social and behavioral challenges manifested in incarceration, loneliness, suicide attempts, and trying to fit in with the prisoner society (a transition). Some of these transitions became major turning points with introduction of drug abuse as part of the individual’s life in prison.

### Professionals: A drug-dealing career

Some of the participants in the present study perceived themselves as professional criminals who had made drug dealing their career:

How much did a construction worker earn back in the seventies? Just a few bucks for a full day's work. When I did that I knew wouldn't make it as a "normal" person, with a "normal" job; it just wasn't for me. I felt I was destined for something better … I couldn't handle the just settling into … into life, that grayness … drug dealing was my way out of the gray life. It was crazy and adventurous, and I made tons of money … After you take that path, you really can't go back to construction … and of course I used, here and there, green mostly, but I was committed so I didn't lose my head … now, close to 60, I still have my health because I was never an addict, but here they treat me like a junkie; they don't get the difference … (IOA, 59).

This participant seemed to take pride in his work in the drug business, reflecting on its benefits and on his dedication to it. Even then, after years of being in and out of prison for drug crimes, he was still enchanted by the “crazy and adventurous” life as a drug dealer who made “tons of money.” This choice “rescued” him from living what he called a “gray life,” mundane and lacking in adventure. The participant did not see himself as a person who abuse drugs and described refraining from drug use as a deliberate act of good business management. However, he was disappointed to learn that, in the prison setting, drug dealing and drugs were all lumped into one category, and he was being treated in the same way as all the other people who use drugs.

Another participant adds:

My thing was never the drug use itself, but yeah, for sure, my life revolved around drugs. I made it, cooked it, sniffed it, delivered it, traveled with it around the world, sold it to poor junkies…Some of the guys in my gang, they fell hard … but not me, everybody uses, but I was never a serious user because this shit can kill you and I'm not that dumb [laughing] … nowadays, I feel compassionate toward junkies, I even try to help them here in prison, offer some of my experience. When I was younger, I just looked down on them … (IOA, 82).

This participant used to be the member of a gang centered around drug manufacturing and dealing. Despite his history of drug use and his assertion that “everybody uses,” he insisted on making a clear distinction between people who use drugs, who are “poor junkies,” and people who deal drugs. It seems that, over the years, he had softened his dismissive agenda toward people who use drugs. At the time of the interview, he was able to relate to them, not as one of them but as a mentor who shared his experiences with them.

These participants used the drug industry to advance themselves economically and professionally in the criminal world. They did not perceive themselves as having a drug problem, and even looked down on others who were dependent on them to deliver the drugs. In that sense, these participants saw themselves as in control of their drug use habits. These findings may be understood using the concepts of agency and control in the LCP. According to the life-course perspective, individuals are active agents in their lives who not only mediate the effect of social structure but also make decisions and set goals that shape the social structure (59). Parallel to this idea is the concept of control cycles whereby individuals modify their expectations and behavior in response to changes in needs or resources, and construct, negotiate, and traverse life-course events and experiences. Thus, participants in the present study perceived drugs as a lucrative resource and viewed their drug involvement as mostly beneficial. This agentic, non-victim-like perception gave them a sense of well-being, despite having spent their aging years in prison, just like other substance abuse victims.

### Lifelong substance abuse: Individuals with a substance abuse disorder who have been using drugs most of their lives

The final theme represents the narratives of individuals who started using drugs either as teenagers or in their early twenties and became individuals with a long-term substance abuse problem as well as recidivist offenders:

This is my fourteenth prison term, all because of drugs. Even when it wasn't directly drugs—it was drugs … every crime I committed, breaking and entering, burglaries—it was all for drug money … I was always broke, slept out on the streets so many times … I wake up today in here and look in the mirror and start crying about all the lost money, about the way I look—like I'm a hundred years old. I was barely conscious for decades and now my time is almost up …(IOA, 64).

This participant described an entire lifetime devoted to drug use, and a criminal career to sustain the flow of drugs. He focused on key resources that were missing from his life in old age, such as youthfulness and a home—and money and time that were wasted on drugs. No longer under the drugs’ captivating power, he became aware of the reality of the losses they had caused.

Another participant narrated as follows:

I moved between boarding schools and foster homes from the age of 9. I could never stay more than a few months before I was thrown out, always the kid who caused too many problems … The only thing back then, the only thing that was kind of constant in my life was drugs. I'm not proud of it, but it was my support, something that was always there to take away the pain, no matter where I went to sleep that day … Now I'm older and smarter, I can say the drugs were like my parents—they put me to sleep at night, they woke me up in the morning … I was an addict by the time I was 15. I didn’t know how to handle basic everyday life without drugs. There were times when I was clean. I even lived with a woman for 3 years and we raised her kids together, but I always went back to drug use … for the last 7 years, I've been living here, in prison, and it's not too bad for me. I'm using drug replacements, I get them regularly here, I have a roof over my head, nice food, even a few friends … (IOA, 60).

This participant described his tragic life story of childhood neglect that seemed to have pushed him into a life of drug abuse and delinquency. He portrayed drugs as loyal companions in the absence of actual human relationships and parental care. The remark “drugs were like my parents—they put me to sleep at night, they woke me up in the morning” exposes the extent of the void in his life, driving him to “adopt” drug use as a parental substitute. Paradoxically, after failing to adapt to life in the community, this participant found a home in prison. Aging in prison seemed to have given him some sense of safety and shelter, as well as adequate health care and drug rehabilitation that had been missing from his life throughout the years.

The participants presenting the current theme described drug use as intertwined with experiences of childhood abuse and neglect and identified it as an inherent part of their identity. LCP postulates that the past has the potential to shape the present and the future, and that early life-course decisions, opportunities, and conditions affect later outcomes. In particular, LCP emphasizes the chain of events or the ripple effect that can begin in early childhood (e.g., parental neglect, going through the foster care system and using drugs to numb the pain). It creates a long-term pattern of cumulative disadvantage (e.g., multiple incarcerations and drug addiction) that is valuable for understanding social inequality in later life, such as that experienced by individuals with lifelong substance abuse problems (60).

## Discussion

The findings of the present study reveal a unique typology of drug-related themes in the lives of IOAs. Whereas for some participants, drugs and drug use formed the background to their criminal thinking (Growing up around drugs), others perceived it as part of prison life (Prison onset). For the rest of the participants, drug use was either a core element in their identity from early childhood (Lifelong substance abuse) or was their main source of income and identity (Professionals). This typology sheds light on the interplay of aging, drug use, and incarceration and the way these three socially marginalized positions may intersect.

The findings of the current study strengthen some of the existing knowledge regarding the origin and onset of drug abuse among older adults. For example, some studies address the familial and environmental background of individuals involved with drug abuse and claim that early exposure to drugs may contribute to engaging in drug use later in life (61). This is in line with the findings revealed in the first theme of the current study, which exemplifies how growing up around drugs may set the stage for future criminal involvement. From a life-course perspective, some studies discuss the possibility of transgenerational transmission of drug abuse tendencies that are a result of both genetic and environmental factors (62, 63). As illustrated by the fourth theme, some IOAs who were involved in drugs from early childhood were trapped in a criminal lifestyle throughout their life course. These participants never had the opportunity to lead a normative life; their fate as recidivist offenders was sealed early on and was compounded by years of drug abuse and addiction. This finding is consistent with a study by Marotta (64), who found that adverse childhood experiences, including exposure to caregiver drug abuse, increased the risk for many substance misuse outcomes among recidivist offenders. In Sweden, Byqvist and Olsson (65) classified male drug users into four groups, the largest of which they called “addicted criminals.” Typical features of this group were criminal activity from an early age, difficult circumstances during childhood, and heavy use of both drugs and alcohol. Thus, the participants’ narratives in the current study seem to tragically portray the life-course principle, according to which the past shapes the future (60).

Contrary to those IOAs who were immersed in the world of drugs from a young age, other IOAs described late-onset drug abuse. This finding highlights the uniqueness of drug use patterns among older adults (16). Late-onset substance abuse accounts for approximately 10% of substance abuse among older adults (66). A number of predisposing factors have been identified for late onset substance abuse, including physical or mental health decline, financial strain, family issues, loneliness, and environmental factors (37, 67, 68). Some of these stress factors are integral to prison life (69, 70). Hence the potential of incarceration to lead to the onset of drug use (71). Moreover, the findings of the current study, as revealed in the second theme, are consistent with other research that reflects the centrality of drug use to prison life (19). Incarceration may be a high-risk environment for substance abuse among adults struggling with the hardships of imprisonment (72). Finally, the third theme, which includes the narratives of older adults who perceived themselves as professional drug dealers, sheds light on another aspect of older adults’ drug abuse patterns. These participants tended to disregard their own drug use habits—consistent with the finding that denial of the drug problem often occurs within the older adult age group (37). Menninger (73) notes that older adults, who grew up at a time when drug abuse was considered a moral weakness and a character flaw, often deny their drug problem because of feelings of shame and guilt. Since denial is a barrier to assessment, diagnosis, and treatment of drug abuse and its effects among older adults, it deepens the problem (74).

In sum, the findings of the current study shed light on the experiences of IOAs and the role of substance abuse in their lives, while highlighting aspects that are unique to this age group and intertwined with life-course factors, such as the power of the past to shape the future, late onset of substance abuse, and denial of the drug abuse problem.

### Limitations and recommendations for further research

The aim of the present study was to explore the different ways in which IOAs narrated their drug abuse experiences. However, to provide a multidimensional understanding of IOAs’ drug abuse, future studies may triangulate between the participants’ own narratives and quantitative data regarding the amount and types of drugs used in different trajectories along the life course. Moreover, this study is limited in its generalization ability due to its small sample size and qualitative nature. Future studies, using quantitative methods, could be helpful for gathering more comprehensive data on this phenomenon. In addition, the participants in the present study volunteered to take part and were eligible if they had the capacity to conduct a meaningful hour-long conversation. Therefore, the experiences of other IOAs affected by drug use, who may have been suffering from psychological and cognitive effects that prevented their participation, are not reflected in the findings. Finally, the study did not differentiate between the participants’ experiences based on drug types and the length and magnitude of abuse. It is possible that abuse of different types may lead to different experiences.

### Practical implications

The research findings pinpoint on the unique interplay between old age, incarceration, and drug abuse. It seems that, in some cases, prison might serve as a nurturing environment in which drug abuse can thrive. Thus, the different ways in which IOAs narrate drug abuse experiences can serve as a framework for developing tailored interventions for IOAs struggling with drug abuse. Finally, hearing the participants’ narratives in their own voices may help professionals relate to the subjective meanings attributed to IOAs’ drug abuse. Understanding the story behind drug abuse and relating to the social and familial trajectories that led to it may prepare the ground for more effective interventions.

## Data availability statement

The datasets presented in this article are not readily available because the data contains restricted information regarding drug use among incarcerated individuals. Requests regarding the datasets should be directed to HA havieli@gmail.com.

## Ethics statement

The studies involving human participants were reviewed and approved by the Ariel University Institutional Review Board. The patients/participants provided their written informed consent to participate in this study.

## Author contributions

The author confirms being the sole contributor of this work and has approved it for publication.

## Conflict of interest

The author declares that the research was conducted in the absence of any commercial or financial relationships that could be construed as a potential conflict of interest.

## Publisher’s note

All claims expressed in this article are solely those of the authors and do not necessarily represent those of their affiliated organizations, or those of the publisher, the editors and the reviewers. Any product that may be evaluated in this article, or claim that may be made by its manufacturer, is not guaranteed or endorsed by the publisher.
